# Risk stratification scheme based on the TNM staging system for dogs with oral malignant melanoma centered on clinicopathologic presentation

**DOI:** 10.3389/fvets.2024.1472748

**Published:** 2024-09-25

**Authors:** Eric Song, Jessica Lawrence, Erica Greene, Anneka Christie, Stephanie Goldschmidt

**Affiliations:** ^1^Apex Veterinary Specialists, Denver, CO, United States; ^2^Department of Surgical and Radiologic Sciences, School of Veterinary Medicine, University of California, Davis, Davis, CA, United States; ^3^RedBank Veterinary Hospital, Tinton Falls, NJ, United States

**Keywords:** oral melanoma, canine, prognosis, oral cancer, malignant, oncology, dogs, OMM

## Abstract

**Introduction:**

Oral malignant melanoma (OMM) is the most common malignant oral neoplasm in dogs. Tumor recurrence, progression, and regional and distant metastasis remain major obstacles despite advanced therapy. Tumor size has been a consistent, key independent prognostic factor; however, other clinical and histopathologic features impact prognosis and likely influence optimal treatment strategies. Adoption of a risk stratification scheme for canine OMM that stratifies groups of dogs on defined clinicopathologic features may improve reproducible and comparable studies by improving homogeneity within groups of dogs. Moreover, it would aid in the generation of multidisciplinary prospective studies that seek to define optimal treatment paradigms based on defined clinicopathologic features.

**Methods:**

To build a platform upon which to develop a risk stratification scheme, we performed a systematic review of clinicopathologic features of OMM, with particular attention to levels of evidence of published research and the quantitative prognostic effect of clinicopathologic features.

**Results:**

Tumor size and presence of bone lysis were repeatable features with the highest level of evidence for prognostic effects on survival. Overall, with strict inclusion criteria for paper review, the levels of evidence in support of other, previously proposed risk factors were low. Factors contributing to the challenge of defining clear prognostic features including inconsistencies in staging and reporting of prognostic variables, incomplete clinical outcome data, inhomogeneous treatment, and absence of randomized controlled studies.

**Discussion:**

To overcome this in the future, we propose a risk stratification scheme that expands the TNM system to incorporate specific designations that highlight possible prognostic variables. The ability to capture key data simply from an expanded TNM description will aid in future efforts to form strong conclusions regarding prognostic variables and their influence (or lack thereof) on therapeutic decision-making and outcomes.

## Introduction

Oral malignant melanoma (OMM) is the most common malignant oral neoplasm in dogs, comprising almost 40% of all oral tumors (1–9). The term OMM refers solely to melanocytic neoplasms originating from the oral cavity that carry aggressive biologic behavior and metastatic potential. This is biologically distinct from histologically well-differentiated melanocytic neoplasm (HWDMN), alternatively referred to as canine oral melanocytic neoplasms of low malignant potential. The distinguishing characteristic between classic OMM and HWDMN is a low mitotic count (< 4/10 mitoses per hpf) combined with a well-differentiated appearance, high pigmentation, and tumor cells with small nuclei that have mild atypia (3, 10). OMM is associated with a high mortality risk due to its propensity for locoregional and distant metastasis (11, 12). Yet, early diagnosis and definitive treatment can yield locoregional tumor control and good clinical outcomes (13).

Staging for dogs with OMM is based on the World Health Organization (WHO) staging scheme, with stage I tumors <2 cm diameter, stage II tumors 2 cm to <4 cm diameter, stage III tumors ≥4 cm diameter, and/or with locoregional lymph node (LN) metastasis, and stage IV tumors with distant metastasis (14). The treatment of choice for canine OMM is wide surgical excision with the intent for histopathologic margins free of neoplasia. Following curative-intent surgery alone, median survival times (MST) may be greater than two years for dogs with stage I and II OMM (13). However, wide ranges in MST have been reported across the literature, with historical literature reporting survival times of 12 months for stage I tumors and less than 12 months for stage II tumors (9, 15). Despite complete excision, the risk of recurrence is 8–17%, potentially due to pitfalls in pathologic processing of specimens, satellite metastasis or field cancerization, and distant metastasis remains a possibility for many dogs (4, 13, 16, 17).

Primary or adjuvant radiation therapy (RT) is integral to the locoregional management of canine OMM (18–32). Although an optimal fractionation scheme is unknown, OMM is radio-responsive, with reported response rates exceeding 80% (21, 24, 30, 32, 33). As with surgery, reported MST spans a wide range from 147–307 days, with reported prognostic factors including the presence or absence of gross disease, stage of disease, tumor location, and infiltration to bone (24, 27, 30–33). Like surgery, better outcomes following RT are reported for dogs with early-stage tumors, with MST up to 758 days for stage I OMM (32). However, the risk of recurrence or progression within the irradiated volume ranges from 15–45% (22, 24, 30). Recent literature supports that dogs with stage I or II OMM treated with surgery and adjuvant RT have improved progression-free survival and overall survival compared to dogs treated with RT alone (30). It is unclear if dogs with stage III or IV OMM have a similar benefit when RT is used postoperatively (30); however, further work is needed to evaluate the importance of combination therapy.

Adjuvant systemic treatment is often utilized, given the high likelihood for OMM to metastasize. Currently available systemic therapies, including chemotherapy and immunotherapy, have not consistently improved outcomes alone or when combined with local therapy (17, 18, 22, 24, 32, 34–39). Most studies have not shown a clear positive impact from the addition of chemotherapy or a commercially available DNA vaccine (ONCEPT®; Boehringer Ingelheim Animal Health USA Inc., Athens, GA, USA) approved for use in stage II and III disease with adequate local control. However, the lack of uniform treatment in veterinary studies may conceal the benefit of systemic therapy for some dogs following adequate local control (18, 37, 39). Because melanoma is an immunogenic tumor (39, 40), much work is ongoing to develop novel immunotherapy agents, including hCSPG4 electrovaccination (16, 40) and canine immune checkpoint inhibitors. Specifically, anti-PD-1, anti-PD-L1, and anti-CTLA4 antibodies can potentially alter treatment paradigms for dogs with advanced OMM in the coming years (41–44). Additional strategies, such as electrochemotherapy, metronomic chemotherapy, tyrosine kinase inhibitors, intralesional chemotherapy or immunotherapy, cytokine treatment, hyperthermia, and others, have also been investigated and are reviewed elsewhere (45).

Regardless of therapy, tumor progression and metastasis pose a serious problem for most dogs with OMM. While the size of the primary tumor has historically been a key prognostic factor, other clinical and histopathologic features also impact prognosis and likely influence optimal treatment strategies (10, 13, 17, 27, 32, 34, 46, 47). One systematic review concluded that histological features, including nuclear atypia score, mitotic count, Ki67 index, and quantification of pigmentation, accurately predicted the likelihood of one-year survival in dogs with OMM (48). Unfortunately, clinical features such as stage, surgical intent, histopathological margin status, tumor location, and bone invasion were not incorporated with histologic features (48).

Prognostic risk stratification based on collective clinical and pathologic features may help guide evidence-based decision-making. For example, determining populations of dogs with OMM that have objective benefits from adjuvant or neoadjuvant therapies following surgery may help prevent suboptimal or unnecessary treatment for some dogs while selecting dogs most likely to benefit (33). Human head and neck mucosal melanoma (HNMM) shares a similar aggressive behavior to canine OMM (1, 11, 49–57). Two staging systems are currently available for HNMM, one specific to HNMM (mmTNM) and one broader system that encompasses primary nasal cavity, paranasal sinus, and oral cavity malignancies (sccTNM). It is currently recommended that both classifications be combined when staging human patients to improve risk stratification and appropriate treatment planning (51, 56). Adoption of a similar risk stratification scheme for canine OMM may allow for improved, reproducible, and comparable groups of dogs for which to build multidisciplinary prospective studies that seek to define optimal treatment paradigms. The aim of this study was to provide a platform upon which to develop a risk stratification scheme. To facilitate this objective, we performed a systematic review of clinicopathologic features of OMM, with particular attention to levels of evidence of published research and the quantitative prognostic effect of clinicopathologic features.

## Materials and methods

A systematic literature search was performed, including articles available through April 31, 2023. The online databases Pubmed, Scopus, and Google Scholar were systematically searched using the terms: [(canine OR dog OR dogs) AND (oral OR mouth OR mucosal) AND (melanoma OR melanocytic) AND (prognosis OR survival OR disease-free interval OR DFI OR outcome)]. De-duplication of articles was performed with Sciwheel,^1^ and title/abstracts were screened for inclusion with Covidence.^2^ Screening was performed independently by a board-certified veterinary dentist and oral surgeon (SG) and a resident in dentistry and oral surgery (ES). Discrepancies on inclusion or exclusion criteria were reached by consensus.

Studies were excluded if they: (1) were not peer-reviewed (2) were not written in English, (3) represented a review or case report, (4) did not differentiate features and prognosis for oral lesions from other sites (e.g., cutaneous, ocular, digital) (5) did not detail clinical prognostic factors and outcomes (6) only included an abstract and not the entire manuscript for review, (7) did not statistically evaluate associations between the specified risk factor and outcome. Outcome data was defined as MST, progression-free interval (PFI), progression-free survival (PFS), disease-free interval (DFI), disease-free survival (DFS), survival probability at a defined time point (e.g., at one or two years), or hazards ratio (HR)/odds ratio (OR) for recurrence, progression, or death.

Data collection was performed by a dentistry and oral surgery resident (ES) and two veterinarians undergoing rotating internship training (EG, AC). Data extracted from the study included the following: number of dogs, study design, specific risk factor(s) evaluated, statistical analysis performed, treatment regimens, prognostic factors, and outcomes. Each study was classified by study design, with levels of evidence applied, as previously described (45) (Table 1). In line with a recent systematic review of veterinary use of mitotic activity, we reported mitotic count, even when prior literature used the term mitotic index (58). Data on each potential risk factor was collated separately for each paper. Data on a specific risk factor was excluded if a risk factor was defined but was not accompanied by numerical data or statistical analysis for prognostic significance. For studies in which both the size (T1-T3) and stage (1–4) were evaluated as risk factors, the size was collected. WHO staging was only applied for studies in which stage was reported without tumor size. Because stage III tumors include criteria for the primary tumor and LN metastatic status, data from studies that did not specify LN status were excluded from analysis because it could not be discerned that stage III tumors had confirmed LN metastasis.

**Table 1:** Format used to grade (A) individual references and (B) the overall level of evidence.

(a) Study type level of evidence (LOE)	Level of evidence
Systematic review (with homogeneity) of randomized controlled clinical trials (RCT)	1a
Individual RCT (with narrow confidence interval)	1b
All or none	1c
Systematic review (with homogeneity) of cohort studies	2a
Individual cohort study (including low-quality RCT; for, e.g., <80% follow-up) or well-controlled laboratory study	2b
“Outcomes” research; ecological studies	2c
Systematic review (with homogeneity) of case–control studies	3a
Individual case–control study or non-randomized controlled clinical trial/study or weak laboratory study	3b
Case series >50 cases	4a
Case series 20 to 50 cases	4b
Case series <20 cases	4c
Case report	4d
Expert opinion without explicit critical appraisal, or based on physiology, bench research or “first principles”	5
(b) Types of Study	Overall evidence grade
Consistent RCT, cohort study, all or none, decision rule validated in different populations	A
Consistent retrospective cohort, exploratory cohort, ecological study, outcomes research, good laboratory study, case–control study, non-randomized controlled clinical trial; or extrapolations from level A studies	B
Case series study or extrapolations from level B studies	C
Expert opinion without explicit critical appraisal, or based on physiology, bench research or first principles	D

Adapted from Pazzi et al. (45).

Data regarding a specific risk factor was combined from all available studies when reported in multiple studies. A combined mean value for MST was manually calculated and presented with a range in parenthesis. Similarly, PFI, PFS, and DFI endpoint data were combined and considered an event-free interval (EFI) to include disease recurrence, progression, or death, and a mean combined EFI was manually calculated and presented with a range in parenthesis.

## Results

**Figure 1:**
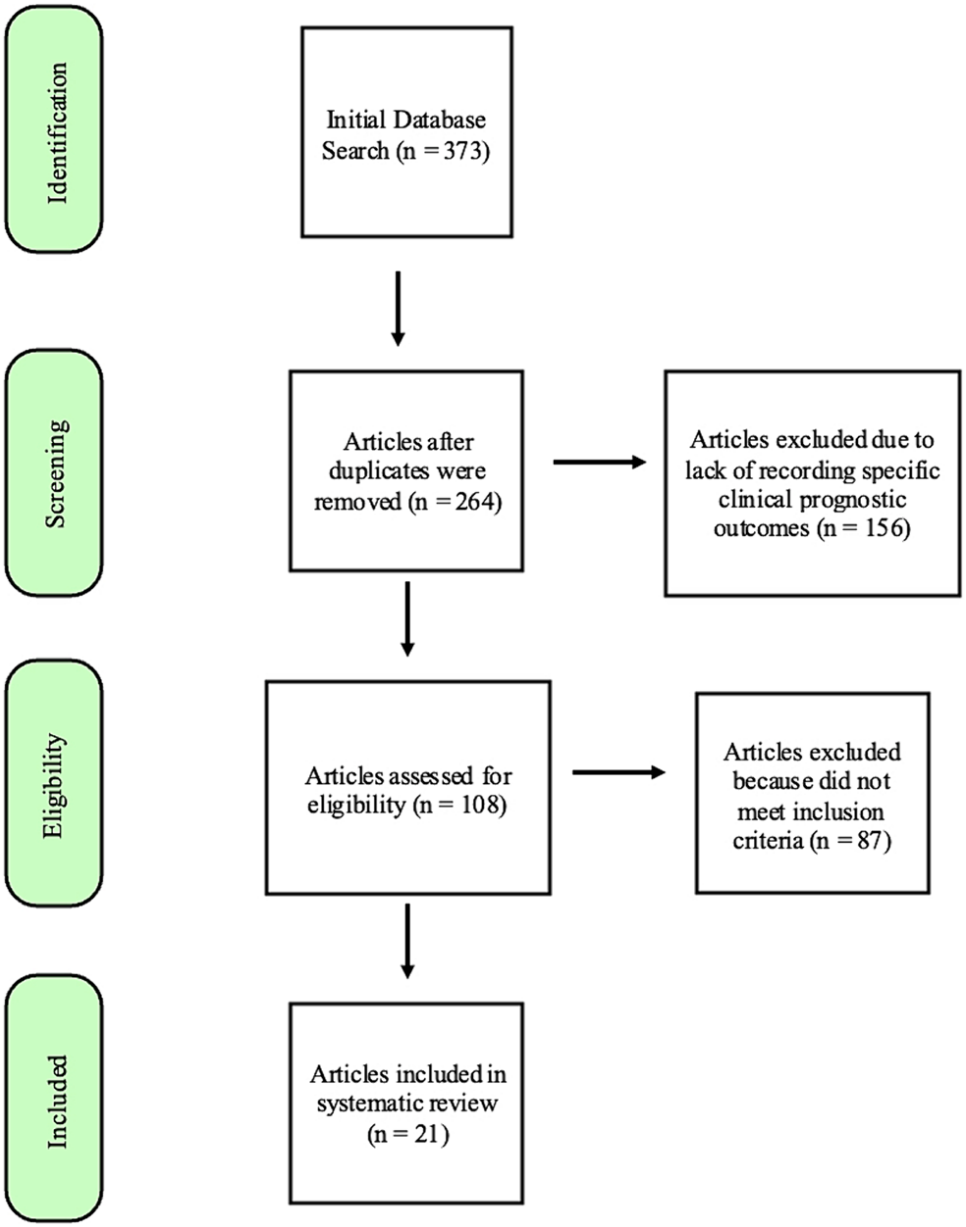
Flowchart modified from the “Preferred Reporting items for Systematic Reviews and Meta-Analysis (PRiSMA) (REF) guidelines,” demonstrating the process by which the search results were narrowed to the 21 articles included in this systematic review.

Database searches provided 373 manuscripts. Twenty-one articles met the inclusion criteria after screening and review (Figure 1). Studies were excluded most commonly because they did not differentiate features and prognosis for oral lesions from other sites or did not sufficiently detail statistical data on associations between the specified risk factor and outcome. Although many manuscripts did not meet inclusion for systematic prognostic review, descriptive data from reviewed manuscripts are also briefly presented in justification of a proposed risk stratification scheme.

### Clinical and tumor-specific features

#### Patient signalment (*N* = 3)

OMM is more common in older dogs, with a mean age between 9 and 12 years (3, 17, 24, 34, 35, 47, 59–65). Poodles, Golden and Labrador Retrievers, Rottweilers, Yorkshire Terriers, Cocker Spaniels, Chow-chows, Scottish Terriers, Pekinese, Gordon Setters, and Dachshunds are overrepresented (2, 13, 59, 60, 63, 66). No studies compare breed prevalence to the hospital population or the relationship between breed and outcome measures.

Three articles provided prognostic information concerning clinical signalment (age and/or sex). Two studies documented age as a significant prognostic factor (17, 32). One retrospective study suggested that dogs <12 years old (*n* = 70) had significantly longer MST (433 days) compared to dogs ≥12 years of age (*n* = 77, MST of 224 days) when treated with surgery with or without adjuvant therapy (17). A second retrospective study (*n* = 111) suggested that increasing age was significantly associated with death within one year following RT (32). Both articles had level 4a evidence and an overall evidence grade of C. Inherited bias could not be controlled for, and it cannot be ignored that age may impact therapeutic decision-making.

Two reports evaluated sex as a prognostic factor (13, 32). One report found sex was not a prognostic factor following RT (32); the other study suggested that intact female dogs had poorer outcomes following surgery (13). This latter study had level 4a evidence; however, there were only two intact females in that study, and a type I error was possible (13). Overall, there is a lack of evidence to support that factors contributing to clinical signalment affect prognosis. Thus, the authors conclude these features should be excluded from risk stratification.

#### Tumor size (*N* = 8)

Evaluating tumor size is the first key clinicopathologic factor to evaluate in dogs with OMM (48). Eight studies evaluated the prognostic influence of tumor size (Table 2). All studies supported that smaller tumors are associated with improved outcomes compared to larger tumors (13, 17, 24, 27, 30, 32, 34, 67).

**Table 2:** Main findings and level of evidence for studies that evaluated tumor size as a prognostic factor for OMM.

Study type	Treatment performed^a^	Number of dogs per tumor size	EFI^b^ (days)	MST (days)	HR	Test type used for significance	Statistical sig (*p* < 0.05)	LOE	Ref
Retrospective	Radiation + Prior Sx (84) + CHMO (80)	Microscopic: 93	285	453	-	Cox proportional-hazards model	Yes	4a	(24)
		Macroscopic: 47	106	158	-				
Retrospective	Surgery + RT (12) + CHMO (32) + metronomic CHMO (4) + Vx (24)	<2 cm: 39	-	630	Reference	Log-rank test and Cox proportional-hazards model, univariate data on HR is presented	Yes	4a	(17)
		2–4 cm: 71	-	240	HR of death: 2.23				
		>4 cm: 25	-	173	HR of death: 5.2				
Retrospective	Vaccine + Sx (105) + RT (71)	<2 cm: 30	-	-	Reference	Cox proportional-hazards model, univariate data on HR is presented	Yes	4a	(34)
		>/= 2 cm: 53	-	-	HR of TTP: 9.15 HR of PFS: 4.59 HR of tumor specific OS:10.26 HR of OS: 4.58				
Retrospective	Surgery + CHMO alone: MTD (7), metronomic (8), both (3) + RT + CHMO: MTD (2), metronomic (4) + CHMO+ Vx or IFN (3) + CHMO, RT + Vx (1) + Vx alone (1)	<3 cm: 23	>567 (NR)	874	Reference	Cox proportional-hazards model, univariate data on HR is presented	Yes (PFI) No (MST)	4a	(13)
		>3 cm: 47	245	396	HR of PFI: 2.0 HR of MST: 1.7				
Retrospective	Radiation + Cytoreductive Sx (21)	< 2 cm: 13	TTR:158	312	-	Log rank test for survival and TTR. Cox proportional-hazards model, multivariant data on HR is presented	Yes for TTR: T1 vs. T3 OS: T1 vs. T3 and T2 vs. T3 No for TTR: T1 vs. T2 OS: T1 vs. T2 HR of TTR and OS	4a	(27)
		2–4 cm: 28	TTR:148	234	-				
		>4 cm: 27	TTR:126	145	HR of tumor size (exact size cutoffs not specified) on TTR: 0.97 on OS: 1.158				
Two randomized, double-blinded trials	Trial 1: 50 dogs were stratified based on clinical stage and randomized to once a week L-MTP-PE or control	Stage 1: 20 L-MTP-PE: 11 Control: 9	DFS: 477 (treatments combined)	NR	-	Breslow and Mantel-Cox tests	Yes	1b	(67)
		Stage 2: 18 L-MTP-PE: 8 Control: 11	DFS:154 (treatments combined)	257	-				
		Stage 3: 11 L-MTP-PE: 6 Control: 5 **	DFS:147 (treatments combined)	226	-				
	Trial 2: 48 dogs were stratified on the basis of clinical stage and extent of surgery (simple resection or radical excision), treated with L-MTP-PE two times a week, and randomized to rcGM-CSF or saline (placebo) given s.c. daily for 9 weeks	Stage 1: 25 L-MTP-PE + rcGM-CSF: 13 L-MTP-PE + saline: 12	DFS:622 (treatments combined)	653	-				
		Stage 2: 15 L-MTP-PE + rcGM-CSF: 7 L-MTP-PE + saline: 8	DFS:118 (treatments combined)	248	-				
		Stage 3: 8 L-MTP-PE + rcGM-CSF: 4 L-MTP-PE + saline: 4 **	DFS:48 (treatments combined)	364	-				
Retrospective	Radiation - orthovoltage (68) - megavoltage (39) - electron beam (4) + debulking Sx (18) + CHMO (39) + Cytoreductive Sx and CHMO (27)	Stage 1: 19	-	758	-	Log rank test followed by Tukey multiple comparisons test	Yes for all stages with Log rank test, on Tukey only between stages I and II and I and III	4a	(32)
		Stage 2: 24	-	278	-				
		Stage 3: 37 dogs **	-	163	-				
Retrospective	Radiation + Curative-intent Sx (24) + Debulking Sx (44) + Vx (77) + CHMO (29)	T1: 13	-	-	RR for OS: Stage 1 vs. 2: 1.39 Stage 1 vs. 3: 0.43 Stage 2 vs. 3: 0.31 Macrosopic versus subclinical: 1.9 RR for PFS: Stage 1 vs. 2: 1.19 Stage 1 vs. 3: 0.40 Stage 2 vs. 3: 0.33 Macrosopic versus subclinical: 2.0 **	Cox proportional-hazards model	Yes for OS for 1 vs. 3, and 2 vs. 3, macrosopic versus subclinical Yes for PFS for 1 vs. 3, and 2 vs. 3, macrosopic versus subclinical	4a	(30)
		T2: 24	-	-					
		T3: 11	-	-					
		Subclinical disease: 19	-	-					

If tumor size in WHO T stage was listed, this was used for analysis. If it was not clarified if stage 3 was due to lymphatic metastasis or tumor >4 cm, the data is presented here rather than in the table evaluating lymphatic metastasis; these cases are marked with a **. ^a^Primary treatment focus of the study is bolded. The number of dogs that received additional treatments are listed in parenthesis. ^b^PFI values reported by default but highlighted as other outcome measure where deviation from PFI occurred. EFI, event free interval; PFI, progression-free interval; DFS, disease-free survival; TTP, time to tumor progression; TTR, time to recurrence; PFS, progression-free survival; HR, hazard ratio; OS, overall survival; MST, median survival time; RT, radiation therapy; Sx, surgery; CHMO, chemotherapy; Vx, vaccine; MTD, maximum tolerated dose; IFN, interferon; L-MTP-PE, liposome-encapsulated muramyl tripeptide. ** Stage 3 not detailed if due to lymph node metastasis or size.

Most studies (*n* = 6) categorized tumor size indirectly using the standard WHO staging scheme. Combined, there were 159 T1 (<2 cm), 181 T2 (2–4 cm), and 119 T3 (> 4 cm) tumors. The combined mean of the reported EFI for T1 tumors was 419 (158–622) days, T2 tumors was 140 (118–154) days, and T3 tumors was 214 (145–364) days. The combined mean of the reported MST for T1 tumors was 589 (312–758) days, for T2 tumors was 251 (234–278) days, and for T3 tumors was 173 (145–364) days. One study that evaluated outcome following surgery was excluded because it stratified tumors by <3 cm or > 3 cm, excluding it from combined analysis (13). Of note, this study reported longer MST (874 days) for tumors <3 cm (*n* = 23) compared to tumors >3 cm (*n* = 47, MST of 396 days). This study also suggested that for each increase in tumor size by 1 cm, there was an associated 32% increased hazard of recurrence or metastasis and a 29% increased hazard of death (13).

Most studies (7/8) (13, 17, 24, 27, 30, 32, 34) provided level 4a evidence to support that tumor size impacts prognosis. One study presented level 1b (67) evidence for this association. Despite an overall low level of evidence (grade C), tumor size is the most repeatable negative prognostic marker for outcome in dogs with OMM. Thus, we recommend incorporating tumor size, using the size guidelines within the WHO staging scheme, during risk stratification.

#### Tumor location within the oral cavity (*N* = 7)

The gingiva is the most common site for canine OMM, with 33–68% of tumors reported as gingival tumors (13, 63, 64, 68–71). There is no clear jaw predilection, with 34–38% reported in the mandible and 23–50% reported in the maxilla (17, 24, 32, 47, 61, 72). Other common locations include buccal mucosa (6–32% of lesions) (13, 32, 47, 63, 64, 69, 71, 72), lips (8.4–24.3% of lesions) (13, 17, 32, 47, 64, 69, 71, 72), tongue (1–14.3% of lesions) (13, 24, 32, 47, 63, 64, 69, 72), palate (1.4–14% of lesions) (13, 17, 24, 32, 47, 64, 69, 71, 72), and tonsil (1.4–2.9% of lesions) (13, 47). Notably, of malignant tonsillar tumors, OMM represents 12% of all neoplasms and is the most likely to metastasize to the tonsil (73).

Seven articles provided information on the prognostic impact of tumor location (13, 17, 24, 27, 32, 34, 70, 74) (Table 3). Three publications categorized tumors into rostral or caudal and defined caudal tumors as distal to the maxillary/mandibular fourth premolar (13, 24, 34, 70). The combined mean of the reported MST for rostral tumors (*n* = 92) was 354 (332–375) days, and for caudal tumors (*n* = 63) was 310 (204–416) days (13, 24, 70).

**Table 3:** Main prognostic findings and level of evidence of studies that evaluated the effect of tumor location on prognosis.

Study type	Treatment performed^a^	Tumor location Number of dogs that had a tumor in each location	EFI^b^ (days)	MST (days)	HR	Test type used for significance	Statistical sig (*p* < 0.05)	LOE	Ref
Retrospective	Radiation + Sx (84)	Rostral: 69	228	331.5	-	Cox regression model, univariate analysis presented	Yes	4a	(24)
		Caudal: 49	140	203.7	-				
Retrospective	Melanoma vaccine + Sx (105) + RT (71)	Rostral: 52	-	-	Reference	Cox proportional-hazards model, univariate data on HR is presented	Yes	4a	(34)
		Caudal: 53			HR of TTP: 2.48 HR of PFS: 2.21 HR of tumor specific OS: 2.64 HR of OS: 2.2				
Retrospective	Surgery + CHMO alone: MTD (7), metronomic (8), both (3) + RT + CHMO: MTD (2), metronomic (4) + CHMO+ Vx or IFN (3) + CHMO, RT + Vx (1) + Vx alone (1)	Rostral: 23	360	375	Reference	Cox proportional-hazards model, univariate data on HR is presented	No	4a	(13)
		Caudal: 14	358	416	HR of PFI: 0.9 HR of MST: 1.2				
Retrospective	Radiation + Cytoreductive Sx (21)	Maxilla: 23 Mandible: 30 Rostral region: 5 Buccal, lip, and hard or soft palate:10	-	-	-	Log rank test for survival and TTR	No	4a	(27)
Retrospective	Radiation - orthovoltage (68) - megavoltage (39) - electron beam (4) + debulking Sx (18) + CHMO (39) + Cytoreductive Sx and CHMO (27)	Maxilla: 40 - Rostral: 29 - Caudal: 9 Mandible: 38 - Rostral: 29 - Caudal: 5 Lip: 11 Buccal mucosa: 11 Hard palate: 4 Soft palate: 4 Tongue: 3	-	Extrapolated from the Kaplan Meier survival curve, dogs with lip and soft palate tumors had improved (not significant) survival compared to dogs with tumors in other locations	-	Log rank test followed by Tukey multiple comparisons test	No	4a	(32)
Retrospective	Pathology study Treatment not specified	Labial mucosa: 6	-	310	-	Not clearly specified	No	4a	(74)
		Lingual: 7 Gingival: 45	-	123	-				
Retro	Surgery + RT (12) + CHMO (32) metronomic CHMO (4) Vx (24)	Mandible: 58 Maxilla: 41 Lip: 34 Palate: 8 Other: 9 Rostral: 53 Caudal: 30	-	-	-	Log-rank test	No	4a	(17)

^aPrimary treatment focus of the study is bolded. The number of dogs that received additional treatments are listed in parenthesis. bPFI values reported by default but highlighted as other outcome measure where deviation from PFI occurred. EFI, event free interval; PFI, progression-free interval; TTP, time to tumor progression; PFS, progression-free survival; OS, overall survival; MST, median survival time; HR, hazard ratio; RT, radiation therapy; Sx, surgery; CHMO, chemotherapy; Vx, vaccine HR, hazard ratio; MST, median survival time; PFI, progression-free interval; OS, overall survival; RT, radiation therapy; Sx, surgery; CHMO, chemotherapy; Vx, vaccine; MTD, maximum tolerated dose.^

Most studies (5/7) have not demonstrated that location within the oral cavity carries a clear prognostic influence on outcome. However, with a broad categorization of rostral and caudal location, two studies have supported that caudal location was associated with poorer EFI and MST compared to tumors in the rostral oral cavity (*n* = 223 combined) (23, 33). Although subcategorization of tumor location has not clearly demonstrated prognostic significance, tumor location impacts therapeutic decision-making, as maxillary and mandibular tumors are more likely to be referred for wide surgical resection, and caudal-location impacts the ability to obtain neoplasia-free margins (75).

There is low level (4a-4b, grade C), contradictory evidence on the effect of oral location as a prognostic factor. Given that the literature to date supports that location is not a clear prognostic factor, the authors recommend that location be excluded from risk stratification at this time and that therapeutic decision-making not be influenced. However, tumor location should be documented separately, with anatomic descriptions to standardize location categorization, in future studies to determine its influence on therapeutic decision-making. Additionally, controlled studies may demonstrate that the location should be incorporated in the risk stratification schema revisions.

#### Bone invasion at presentation (*N* = 6)

Twelve of the screened studies reported the presence of bone invasion. Of OMM included, 28–92% of tumors presented with bone invasion at diagnosis (9, 16, 22, 24, 27, 33, 34, 47, 60, 65, 76, 77). Not all tumors were imaged before treatment, and imaging techniques varied from standard radiography to contrast-enhanced CT, limiting conclusions on the true prevalence of bone invasion.

Six studies provided prognostic significance of bone invasion secondary to the primary OMM (24, 27, 34, 47, 60) (Table 4). Combining data from these studies, 45% of OMM present with bone invasion (*n* = 159), and 55% do not (*n* = 194). All but one study supported that bone invasion at presentation is a significant negative prognostic indicator. The combined mean of the reported EFI for dogs with bone lysis was 240 (72–193) days compared to 271 (164–470) days for dogs without lysis. The combined mean of the reported MST following treatment for dogs with bone invasion was 217 (117–397) days compared to 522 (246–1,063) days for dogs without bony lysis.

**Table 4:** Main prognostic findings and level of evidence of studies that evaluated the effect of bone lysis on prognosis.

Study type	Treatment performed^a^	Presence of bone lysis Number (percent with lysis at presentation)	EFI^b^ (days)	MST (days)	HR	Test type used for significance	Statistical sig (p < 0.05)	LOE	Ref
Retrospective	Surgery + CSPG4 EVT ECT (5) CHMO (26)	Bone lysis: 28 (41%)	DFI: 193	397	-	Log-rank test	Yes	4a	(47)
		No lysis: 40 (59%)	DFI: 470	1,063	-				
Retrospective	Radiation + Cytoreductive Sx (21)	Bone lysis: 39 (57.3%)	TTR: 104	139	-	Log rank test for survival and TTR	Yes	4a	(27)
		No lysis: 29 (42.7%)	TTR:164.5	259	-				
Retrospective	Radiation + CHMO (80)	Bone lysis: 31 (28%)	73	118.6	-	Cox regression model	Yes	4a	(24)
		No lysis: 80 (72%)	182.5	249	-				
Longitudinal prospective study	ECT	Bone lysis: 42 (64.6%)	-	-	HR of OS: 2.45 HR of TTP: 0.42	Log-rank test and Cox multiple regression model, univariate results presented	Yes	3b	(60)
		No lysis: 23 (35.4%)	-	-	Reference				
Retrospective	Melanoma vaccine + Primary Sx (105) + Revision Sx (7) + RT (71)	Bone lysis: 19 (35%)	-	-	HR of tumor specific OS: 2.26 HR of OS: 1.94 HR of TTP: 2.5 HR of PFS: 2.12	Cox proportional-hazards model, univariate data on HR is presented	Yes	4a	(34)
		No lysis: 35 (65%)	-	-	Reference				
Retrospective	Radiation + Curative-intent Sx (24) + Debulking Sx (44) + Vx (77) + CHMO (29)	Not specified	-	-	RR for OS: 1.15 RR for PFS: 1.38	Cox proportional-hazards model	No	4a	(30)

^aPrimary treatment focus of the study is bolded. The number of dogs that received additional treatments are listed in parenthesis. bPFI values reported by default but highlighted as other outcome measure where deviation from PFI occurred. EFI, event free interval; PFI, progression-free interval; DFI, disease-free interval; TTP, time to tumor progression; TTR, time to recurrence; PFS, progression-free survival; HR, hazard ratio; OS, overall survival; MST, median survival time; RT, radiation therapy; Sx, surgery; CHMO, chemotherapy; Vx, vaccine; MTD, maximum tolerated dose; ECT, electrochemotherapy; EVT, electrovaccination.^

There is a low level (3b-4a, grade C) of evidence for the role of bone invasion as a risk factor. Yet, all but one study (30) suggests bone invasion is a negative prognostic factor for outcome. Because bone invasion increases risks associated with local tumor control and has repeatedly negatively affected outcome measures, the presence or absence of bony lysis should be included in risk stratification.

### Biologic behavior

#### Lymphatic metastasis at presentation (*N* = 5)

Seventeen of the screened studies reported LN metastatic rate. Collectively, 9–69% of local LN were metastatic at diagnosis (4, 9, 13, 17, 24, 30, 32–34, 47, 70, 77–82). Variable screening techniques (palpation, diagnostic imaging, cytology, pathology) and the number of LN tested (ranging from the ipsilateral mandibular node to complete bilateral neck dissection) were reported, limiting concrete conclusions regarding the metastatic risk of OMM to regional LN.

Five studies evaluated statistical prognostic information on the impact of lymphatic metastasis (Table 5). The combined mean of the reported EFI for dogs with LN metastasis was 150 (112–187) days compared to 386 (206–567) days for dogs without LN metastasis (13, 24). The combined mean of the reported MST for dogs with OMM and LN metastasis was 150 (131–170) days compared to 526 (234–818) days for dogs without confirmed LN metastasis (13, 24, 70). There was no consensus as to the impact of LN metastasis on outcome. Studies were divided on whether LN metastasis negatively impacted prognosis (*N* = 2) (13, 34, 70) or whether LN metastasis did not impact outcome measures (*N* = 3) (17, 24, 33).

**Table 5:** Effect of lymphatic metastasis at presentation on prognosis.

Study type	Treatment performed^a^	LN staging performed with:	LN met rate	EFI^b^ (days)	MST (days)	HR	Test type used for significance	Statistical sig (*p* < 0.05)	LOE	Ref
Retrospective	Radiation + CHMO (80)	MLN cytology	59% (72/122)	N+: 112 N0: 206	N+: 170 N0: 234	-	Cox regression model, univariate analysis presented	No	4a	(24)
Retrospective	Surgery + RT (12) + CHMO (32) metronomic CHMO (4) Vx (24)	MLN cytology (62) or pathology (95)	Cytology: 45.1% (28/62) Histopathology: 58.9% (56/95)	-	-	-	Log-rank test	No	4a	(17)
Retrospective	Melanoma vaccine + Primary Sx (105) + Revision Sx (7) + RT (71)	Cytology and/or pathology performed: Bilateral MLN and MRLN (1) Bilateral MLN (38) Combination of a subset of MLN MRLN (3) Single MLN (66)	26.7% (35/131) on cytology and/or histopathology	-	-	HR of TTP: 2.35 HR of PFS: 2.03 HR of tumor specific OS: 2.34 HR of all-cause mortality: 2.06	Cox proportional-hazards model, univariate data on HR is presented	Yes	4a	(34)
Retrospective	Radiation	MLN cytology	16.7% (3/18)	-	-	-	Pearson and Spearman correlation coefficients	No	4c	(33)
Retrospective	Surgery + CHMO alone: MTD (7), metronomic (8), both (3) + RT + CHMO: MTD (2), metronomic (4) + CHMO+ Vx or IFN (3) + CHMO, RT + Vx (1) + Vx alone (1)	MLN cytology	11.4% (8/70)	N+: 187 N0: 567 **	N+: 131 N0: 818 **	HR of PFI: 3.9 HR of MST: 5.1 **	Cox proportional-hazards model, univariate data on HR is presented	Yes	4a	(13)

^a^Primary treatment focus of the study is bolded. The number of dogs that received additional treatments are listed in parenthesis. ^b^PFI values reported by default but highlighted as other outcome measure where deviation from PFI occurred. EFI, event free interval; PFI, progression-free interval; TTP, time to tumor progression; PFS, progression-free survival; HR, hazard ratio; OS, overall survival; MST, median survival time; RT, radiation therapy; Sx, surgery; CHMO, chemotherapy; Vx, vaccine; MTD, maximum tolerated dose; ECT, electrochemotherapy; LN, lymph node; MLN, mandibular lymph node; MRLN, medial retropharyngeal lymph node; PTLN, parotid lymph node; HOD, hazard of death. **This EFI and MST analysis was for metastasis at diagnosis, which also included 1 patient with pulmonary metastasis.

LN screening and treatment were variable among the five studies. All reported mandibular lymph node cytology, with three studies relying solely on this approach (13, 24, 33). The combined mean of the reported metastatic rate determined by cytology was 30.9 (11.4–59) %, and by histopathology was 37.5 (16–58.9) % (13, 17, 24, 33, 34, 70). Two studies evaluated LN metastasis by both cytology and pathology (17, 34, 70); however, only one article (17) differentiated the metastatic rate based on cytology (45.1%) versus histopathology (58.9%).

Based on the studies included in this systematic review, there is a low level of evidence (4a-4c, grade C) supporting that LN metastasis influences prognosis, and there is conflicting evidence to this point. However, LN screening for many dogs may have been incomprehensive, thus missing metastatic LNs. Further confounding the literature is that stage III disease is associated with a poorer outcome than stages I and II. Because LN metastasis and dogs with large tumors and lack of LN metastasis are included within this staging group, further investigation is warranted to isolate the independent prognostic significance of LN status. The identification of LN metastasis needs to be consistent and sensitive, and treatment for dogs with LN metastasis needs to be standardized in future studies so reliable conclusions can be generated regarding their prognostic influence. This is especially true for cases of micro versus macro-metastasis and single versus multiple metastatic lymph nodes. In humans with malignant melanoma, LN metastasis is a negative prognostic factor and directly influences therapeutic decisions and prognosis (50–52, 54–57, 83–90). Taking into account the following factors: known uncertainties with imaging, confirmation of LN metastasis (or lack thereof) in most of the reported studies, the knowledge of locoregional metastatic prognostic implications in humans, and the recent expert-based canine consensus statement (75), LN status, including metastatic degree and number of LN affected, should be incorporated within the risk stratification scheme.

#### Distant metastasis at presentation (*N* = 4)

Excluding studies that purposely did not include dogs with stage IV OMM, eight reported the distant metastatic rate at presentation. Pulmonary metastasis was reported in 1.4–26% of patients, and abdominal metastasis in 0–5.3% (13, 17, 24, 30, 32, 60, 81, 91). Variable screening techniques were reported, limiting conclusions on OMM’s true distant metastatic risk. Further, because pulmonary metastasis may have been identified on thoracic imaging (e.g., radiographs), abdominal imaging may not have been performed to identify true abdominal metastasis. Indeed, thoracic radiographs were performed exclusively in three studies (13, 24, 81) and combined with thoracic computed tomography (CT) in three studies (17, 30, 91). Abdominal imaging was performed in two studies (30, 91). Both abdominal ultrasound and CT were used in one study (30), and only abdominal ultrasound was used in the other (91). When metastatic rate was specified by which imaging modality was used, pulmonary metastasis was diagnosed in a combined mean of 5.2% (*n* = 19/352; range 1.4–26%) (13, 17, 24, 81) and 6.5% (*n* = 16/246; range 5.7–16.7%) (16, 17, 91) by thoracic radiographs or CT, respectively.

Four studies reported the prognostic significance of distant metastasis at presentation (13, 24, 30, 32, 60) (Table 6). Most (3/4) studies concluded that distant metastasis was a negative prognostic factor (13, 30, 32, 60). Two studies reported the metastatic rate and MST time in their cohort; one had EFI data. The mean of the reported EFI for dogs with stage IV OMM (*n* = 11) was 120 days compared to 220 days for dogs without distant metastasis (*n* = 67) (13, 60). The combined mean of the reported MST for dogs with stage IV OMM (*n* = 42) was 115 (80–135) days compared to 562 (225–900) days for dogs with stage I-III OMM (*n* = 135) (13, 32, 60).

**Table 6:** Main findings and level of evidence of studies that evaluated the effect of distant metastasis on prognosis.

Study type	Treatment performed^a^	Thoracic staging performed with: Abdominal staging performed with:	Pulmonary met rate Abdominal metrate	PFI (days)	MST (days)	RR	Test type used for significance	Statistical sig (*p* < 0.05)	LOE	Ref
Retrospective	Radiation + Chemotherapy (80)	Thoracic Radiographs N/A	7% (9/138)	-	-		Cox regression model, univariate analysis presented	No	4a	(24)
Retrospective	Radiation + Curative-intent Sx (24) + Debulking Sx (44) + Vx (77) + CHMO (29)	Thoracic Radiographs (66) Thoracic CT (32) AUS (13) Abdominal CT (26)	4% (4/98) 0% (0/39)	-	-	RR for PFS: Stage 1 vs. 4: 0.23 Stage 2 vs. 4: 0.27 Stage 3 vs. 4: 1.05 RR for OS: Stage 1 vs. 4: 0.12 Stage 2 vs. 4: 0.07 Stage 3 vs. 4: 0.36	Cox proportional-hazards model	Yes	4a	(30)
Retrospective	Radiation - orthovoltage (68) - megavoltage (39) - electron beam (4) + debulking Sx (18) + CHMO (39) + Cytoreductive Sx and CHMO (27)	Head and Thoracic CT (111) N/A	28% (31/111)	-	Stages 1–3: range from 163–758 (mean: 460) Stage 4: 80	-	Log rank test followed by Tukey multiple comparisons test	Yes between stages I and IV	4a	(32)
Longitudinal prospective study	ECT	Thoracic radiographs AUS	16.4% (11/67)	Stage 1: 11 (range 4–30) months Stage 2: 7 (range 3–21) months Stage 3: 4 (range 2–4) months Stage 4: 4 (range 1–4) months	Stage 1: 16.5 (range 4–30) months Stage 2: 9 (range 4–21) months Stage 3: 7.5 (range 3–17) months Stage 4: 4.5 (range 2–7) months	HR OS: 0.29 HR TTP: 0.34	Log-rank test and Cox multiple regression model, univariate results presented	Yes	3b	(60)

^aPrimary treatment focus of the study is bolded. The number of dogs that received additional treatments are listed in parenthesis. bPFI values reported by default but highlighted as other outcome measure where deviation from PFI occurred. MST, median survival time; PFI, progression-free interval; OS, overall survival; RT, radiation therapy; Sx, surgery; CHMO, chemotherapy; Vx, vaccine; MTD, maximum tolerated dose; IFN, interferon; CT, computed tomography; AUS, abdominal ultrasound; RR, risk ratio; ECT, electrochemotherapy.^

Overall, there is a low level (3b-4a, grade C) of evidence for the role of distant metastasis as a negative prognostic factor. Yet, there is a consistent negative prognostic implication in all studies (29, 31, 60) except for one (23), and survival ranges are generally narrow for dogs with stage IV OMM. Further, given the lack of a clear role for adjuvant chemotherapy and immunotherapy, the presence of distant metastasis negatively impacts prognosis. Thus, the presence or absence of metastasis should be included in risk stratification.

### Pathologic features of the tumor

#### Ki67 (*N* = 4)

Four studies evaluated the prognostic significance of Ki67 (47, 62, 92, 93) (Table 7). The most common cutoff reported for Ki67 proliferation value was 19.5% (*n* = 2). Combined data cannot be presented, as outcomes were not standardized. One study demonstrated an overall survival of 484 days with Ki67 < 19.5% versus 224 days for those with Ki67 ≥ 19.5% (62).

**Table 7:** Main findings and level of evidence of studies that evaluated the effect of pathologic features on prognosis.

Type of study	Number of samples	Prognostic outcome evaluated	Test type used for significance	Statistical sig (*p* < 0.05)	Cutoff value	Outcome	LOE	Ref
Ki67								
Retrospective (62)	48	MST and DFI	Cox proportional-hazards model	Yes	> 19.5%	DFI - 188 days OS - 224 days	4b	(62)
					< 19.5%	DFI - 484 days OS - 484 days		
Retrospective	80	Death within 1 year of diagnosis due to melanoma vs. still alive or death due to other cause	Nonparametric Mann–Whitney U Test	Yes	Mean: 11.2%	Alive at 1 year	4a	(92)
					Mean: 40.8%	Death at 1 year if Ki67 ≥ 19.5%: Sensitivity: 87.1% Specificity: 85.4%		
Retrospective	37	MST	Log-Rank test	Yes	Higher Ki67 (not specified)	125 days	4b	(93)
					Lower Ki67 (not specified)	218 days		
Retrospective	68	MST	Spearman’s correlation	Yes (only for OMMs with bone invasion)	19.5%	MST and Ki67 (*p* = 0.02, r = −0.43) were only significantly correlated in OMMs with bone invasion.	4a	(47)
Mitotic count								
Retrospective	80	Death within 1 year due to melanoma	Nonparametric Mann–Whitney U Test and Cox proportional-hazards model	Yes	MC: ≥ 4/10 hpf	Death at 1 year: Sensitivity: 90.3% Specificity: 84.4%	4a	(92)
Retrospective	68	MST	Spearman’s correlation	Yes (only for OMMs with bone invasion)	MC: 4/10 hpf	MST and MI (*p* = 0.04, r = −0.39) were only significantly correlated in OMMs with bone invasion.	4a	(47)
Retrospective	50	MST	Student’s t-test and one-way ANOVA	No	MC: > 3/10 hpf	No statistically significant differences in survival of dogs with tumors with a high MC (> 3) compared to those with a MC ≤ 3 (*p* = 0.15)	4b	(71)
Retrospective	63	MST	One-way ANOVA	No	MC: > 3/10 hpf	MC not associated with survival	4a	(36)
Retrospective	131	MST	Cox proportional-hazards model	No	MC: ≥ 4/10 hpf or > 3/10 hpf	MC negatively correlated with outcome in univariate analysis MC no effect on outcome in multivariable analysis	4a	(34)
Retrospective	69	-	Log-rank test	No	MC: 4/10 hpf	MC was not a statistically significant prognostic factor.	4a	(38)
Retrospective	27	MST	Log rank tests	No	MC: did not specify cutoff	MC (*p* = 0.1604) not an independent indicator for survival of dogs with OMM	4b	(94)
Retrospective	64	OS and PFS	Cox proportional-hazards model	No	MC: 4 /10 hpf	RR OS: 0.73, RR PFS: 1.8	41	(30)
Nuclear atypia								
Retrospective	80	Death within 1 year due to melanoma	Nonparametric Mann–Whitney U Test and Cox proportional-hazards model	Yes	NA: >30%	Death at 1 year: Sensitivity: 83.9% Specificity: 86.0%	4a	(92)
Retrospective	50	MST	Student’s t-test and one-way ANOVA	No	Cellular atypia: high ≥4	There were no statistically significant differences in survival of patients with tumors with a cut of > or < 4 *p = 0.62	4b	(71)
Retrospective	27	MST	Log rank test	Yes	NA: ≥ 5 (high)	Correlated negatively with post-surgical survival (*p* = 0.0008, r = 0.6153). Higher NA was observed in MM of dogs that died due to the disease compared with those that were censored (*p* = 0.0103), as well as in dogs that survived for <1 year after surgery compared with dogs with longer survival (*p* = 0.0002).	4b	(94)
					NA: ≥ 4	Kaplan Meier survival analysis revealed shorter survival for the group with higher nuclear atypia (median survival = 173 days) than for the group with low atypia (median survival = 486 days, *p* = 0.0004)		
Pigmentation								
Retrospective	80	Death within 1 year due to melanoma	Nonparametric Mann–Whitney U Test and Cox proportional-hazards model	No	Pigmentation: no, low, moderate, high	Death by 1 year Pigment threshold for sensitivity and specificity >75% could not be identified	4a	(92)
Retrospective	27	MST	Log rank test	No	Not specified	Pigmentation (*p* = 0.5991) poor indicator for survival of dogs with OMM	4b	(94)
Retrospective	69	MST	Log-rank test	No	Pigmentation: >50%	508 days	4a	(38)
					Pigmentation: <50%	310 days The degree of pigmentation is not a statistically significant prognostic factor.		
Case series (not retrospective)	16 melanotic 9 amelanotic	MST	Student’s t-test	Yes	Amelanotic vs. melanotic	MST were significantly different between the two melanoma types. MC significantly lower in melanotic melanomas (*p* < 0.01)	4b	(95)

MST, median survival time; DFI, disease-free interval; OS, overall survival; MI, mitotic index; hpf, high power field; MC, mitotic count; NA, nuclear atypia.

There is an overall low level (4a-4b, grade C) of evidence that Ki67 is an independent negative prognostic factor for outcome. Given the small number of studies upon which to form a conclusion yet the lack of contradictory data, Ki67 may be valuable in risk stratification. Additional studies are needed to verify its independent pathologic prognostic significance.

#### Mitotic count (*N* = 8)

Eight studies evaluated the statistical prognostic implication of the mitotic count (34, 36, 38, 47, 70, 71, 92, 94) (Table 7). The most common cutoff for the mitotic count was <4/10 per high-power field [hpf] (5/8 publications). Most (6/8) studies concluded that the mitotic count was not independently associated with outcome measures when evaluated as a single variable (34, 36, 38, 70, 71, 94).

Overall, there is a low level (4a-4b, grade C) and contradictory evidence on the role of mitotic count. Despite this, mitotic activity should continue to be captured in risk stratification, and studies reporting both Ki-67 and MC are needed to better discern the value of each variable in the future.

#### Nuclear atypia (*N* = 3)

Three studies evaluated nuclear atypia score as a prognostic factor (71, 92, 94) (Table 7). The cutoff value was not standardized across the studies. Two studies concluded that the nuclear atypia score was statistically significant with clinical outcome (93, 95), while the remaining study found no association with outcome (71).

Overall, based on the studies included, there is a low level (4a-4b, grade C) of contradictory evidence on the role of nuclear atypia as a prognostic indicator. However, the majority support its inclusion into risk stratification, and the oncology-pathology working group have strongly endorsed its routine inclusion into pathologic prognostication for OMM (48). Thus, nuclear atypia should be considered in risk stratification.

#### Pigmentation (*N* = 4)

Four studies evaluated the degree of tumor pigmentation as a prognostic factor (38, 92, 94, 95) (Table 7). The degree of pigmentation used as a cutoff value was not standardized across the studies. One study demonstrated that pigmentation was associated with a survival advantage, as melanotic tumors had improved outcomes compared to amelanotic tumors (95). The remaining three studies did not demonstrate a clear survival advantage for dogs with pigmented tumors compared to dogs without (38, 92, 94).

Overall, there is a low level (4a-4b, grade C) of evidence supporting that the degree of tumor pigmentation significantly affects prognosis. Further, most studies do not support that it has significant prognostic value, and there is no clear cut-off for pigmentation. Therefore, we recommend that the degree of pigmentation be excluded from risk stratification at this time.

### Other potential pathologic and clinical prognostic factors

Numerous other publications have recently emerged investigating pathologic and clinical features that may be associated with clinical outcomes. Of interest, tumor-infiltrating lymphocytes (TIL) were shown to affect prognosis, with a higher survival rate with higher TIL scores, a brisk or non-brisk TIL pattern, and an increased frequency of CD8+ T lymphocytes infiltrating the tumor (71). Similarly, one study highlighted that a higher frequency of regulatory T lymphocytes, both infiltrating and surrounding the tumor, was associated with poorer PFS and MST compared to dogs with lower frequency (71). Co-expression of *α* and *β* PDGFR and lower serum and plasma VEGF concentrations have been associated with a negative impact on prognosis (62, 96). Interestingly, serum and plasma VEGF concentrations were associated with survival in dogs that received curative-intent therapy but not in dogs that received palliative therapy (6). Dogs with a C-reactive-protein–albumin ratio (CRP/ALB) > 1.9 before tumor excision have been shown to be associated with shorter PFS and MST (97). Lastly, one study explored the prognostic value of somatic focal amplifications on chromosomes (*Canis Familiaris* [CFA]) 10 and 30 in canine OMM (98). This study concluded that CFA 30 amplification was significantly associated with a worse prognosis and correlated with amelanotic phenotype and high mitotic count. Many of these recently reported features may indeed contribute to risk stratification for therapeutic paradigms, such as tumor-infiltrating immune cells and immune checkpoint protein expression. Future work must evaluate inclusion into adapted versions of a risk stratification scheme.

### Proposed risk stratification scheme

Based on the overall low level of evidence that supports that specific risk factors in canine OMM impart an objective negative impact on outcome, additional data is needed to solidify the impact of these risk factors. The WHO TNM system used for staging is routinely utilized in veterinary staging, and to capture data in future studies and systematic analyses, we propose an adaptation to the system (Table 8) that incorporates tumor-related features suggested to impact outcome, extent of lymph node involvement, and highlights what presumptions may be made during staging. Designations are also provided given that not all dogs may be fully staged or may not have confirmation of LN and distant metastasis. The key data collected with use of this scheme strengthens conclusions that can be made following interventional studies, and transparently acknowledges missing data. Examples for how use of the scheme may improve data used for studies is presented in Supplementary Table S1. Incorporation of this scheme into clinical practice, and adapted versions in the future, could be readily accomplished with use of a standardized worksheet (Supplementary Table S2), similar to the stomatitis disease activity index score (99).

**Table 8:** Suggested risk stratification for OMM.

Tumor		Description	T Stage
Tumor size		<2 cm	T1
		2–4 cm	T2
		≥ 4 cm	T3
Tumor designations			
Presence of bone invasion		No	a0
		Yes	a1
Mitotic activity			
Ki67	Mitotic count		
<19.5	< 4	Low	b0
>19.5	≥ 4	High	b1
If discordant, utilize Ki67 proliferative value			
Nuclear atypia		<30%	c0
		>30%	c1
Additional designations			
Tumor feature is unknown			*
Tumor stage summary			T1-T3_a0–1b0-1c0-1_
Lymph node		Description	N Stage
Lymph node metastasis		None	N0
		Micrometastasis (< 2 mm) in a single node	N1
		Macrometastais (> 2 mm) in a single node	N2
		Macrometastasis with extranodal extension in a single node	N3
		Multiple metastatic (micro or macro) lymph nodes	N4
Lymph node designations			
LN detected by sentinel lymph node guided biopsy/cytology			a
Metastatic disease is cytologically confirmed			c
Metastatic disease is presumptive based on imaging only			i
Lymph node status is unknown			*
Lymph Node Stage Summary			N*-0-N4_aci_
Distant metastasis		Description	M stage
Evidence of distant metastasis		Absent	M0
		Present	M1
Metastasis designations			
Distant metastatic status is unknown			*
Metastatic disease detected on radiographs			r
Metastatic disease is cytologically or histologically confirmed			p
Distant Metastasis Stage Summary			M*-M0-M1_rp_

## Discussion

The primary objective of this study was to systematically evaluate levels of evidence and quantitative effects of canine OMM clinicopathologic risk factors on outcome to develop a risk stratification scheme for therapeutic decision-making across specialties. Despite OMM being the most investigated oral tumor in veterinary literature, when strict inclusion criteria were selected, the levels of evidence in support of proposed risk factors were low. Factors including inconsistencies in staging and reporting of prognostic variables, incomplete clinical outcome data, inhomogeneous treatment, and absence of randomized controlled studies contributed to these low levels of evidence. In fact, only tumor size was associated with level 1b evidence to support a negative impact on outcome, whereas most studies included provided level 4a or 4b evidence.

Notably, our levels of evidence in support of risk factors for canine oral OMM were weaker than those reported in a recent report describing international consensus and guidelines for therapeutic decision-making (75). The narrow inclusion criteria in our study which required statistical evaluation of prognostic risk factors coupled with the inherent limitations of retrospective veterinary studies likely contributed to our low levels of evidence. However, it is clear from both studies that overall, the levels of evidence used to derive conclusions for canine OMM are low, rarely reaching level 1 or 2.

There are several gaps in reported literature pertaining to both tumor and staging features that can be captured in the future to strengthen associations between prognostic factors, and aid in stratifying dogs for treatment for optimal benefit. Therefore, we propose use of a risk stratification scheme based on the TNM staging system to ease collection of pertinent data. This scheme can be applied prospectively and retrospectively, and may lead to improved ability and quality of comparisons between publications and more uniform data collection. Like the goals associated with eight edition of the American Joint Committee on Cancer (AJCC) human malignant melanoma staging system, the aim is to facilitate accurate risk stratification and guide therapeutic approaches (100). The proposed scheme incorporates several designations to aid in extraction of specific TNM data for analysis in future studies and acknowledges that not all dogs included in a future study will have complete data regarding disease status. The risk stratification scheme is not intended to discourage additional investigation of the presence or strength of risk factors not included, like histopathologic margin distance, peritumoral or intratumoral T lymphocyte subset infiltration, or tumor location. Rather, it is currently focused on risk factors with repeatable prognostic implications based on systematic review, with the aim to be adapted in the future as knowledge increases.

Currently, tumor size and bone invasion were the most repeatable tumor-related features associated with clinical outcomes. Tumor size has long been a key prognostic feature within the existing TNM staging system, with one study demonstrating a clear increased hazard of death with every 1 cm increase in tumor size (13). Unexpectedly, lymph node metastasis, which is an identified parameter of the TNM system, was not strongly supported as a negative risk factor in the studies included. While limitations including poor power and incomplete staging of LN metastasis in prior studies contributed to the inability to define a clear negative influence of LN metastasis on outcome measures, the presence or absence of LN metastasis clearly influences clinical therapeutic decision making (75, 101). The current TNM definition of stage 3, which includes dogs with tumors equal to or greater than 4 cm in diameter with dogs with lymph node metastasis regardless of tumor size or extent of metastasis (micro- or macro-metastasis) contributes to difficulties in interpreting associations between staging features and outcome. Specifically, the use of stage 3 to classify OMM dilutes the ability to determine the true prognostic effect of both large tumors and metastatic lymph nodes.

Establishing the true impact of LN metastasis is also difficult as prior studies may include dogs that are under-staged or those with presumptive metastasis based on palpation or imaging alone. Methods for LN screening and confirmation of LN metastasis have evolved in recent years to better capture known metastasis (61, 77, 78, 102–104). Lymphatic drainage within the canine head is complex, with multiple lymphocentrums including the parotid, mandibular, and medial retropharyngeal lymph centers. It has been shown that up to 62% of oral tumors have contralateral dissemination (79) and that 6–45.5% of neoplasms spread to lymphocentrums other than the mandibular LNs (80, 82, 105). Yet, only the lateral mandibular LNs are readily accessible without ultrasound guidance; indeed, three of the six critically reviewed studies included here with prognostic data pertaining to LN metastatic status relied only on mandibular LN cytology for staging, often only including the ipsilateral side (13, 24, 33). Cytology has moderate accuracy for canine cervical LNs but may not capture all metastatic LNs (105–107). Several studies have evaluated all six regional lymph nodes via histology in dogs with OMM to determine drainage and metastatic patterns (9, 108–110). However, the need for pathologic staging of all six LNs in dogs without presumptive overt metastasis (the N0 neck) remains controversial. Sentinel LN (SLN) mapping provides an alternative, lower-morbidity option (103, 104, 111, 112) in which to guide cytologic or histopathologic sampling.

Further, the current TNM staging system does not account for differences in the extent of cervical metastasis, which limits conclusions on how to stratify treatment in these patients. In humans, there is a distinct prognostic difference for melanoma between isolated tumor cells, micrometastasis (< 2 mm), macrometastasis, and extranodal extension (100). The number of positive LN is also directly related to tumor burden and clinical outcome (113–117), further solidifying the importance of comprehensive staging. Yet, treatment that results in complete nodal dissection or irradiation of the entire lymphatic basin should ultimately be influenced by the type and number of positive lymph nodes, reducing indiscriminate nodal extirpation or elective irradiation. Historically, human patients with melanoma at risk for nodal metastasis underwent SLN biopsy, with complete LN dissection in the event of a SLN positive for metastasis (118, 119). However, while local disease recurrence was reduced by this approach, there was no difference in survival compared to patients that did not undergo complete LN dissection (118, 119). Similarly, the addition of adjuvant hypofractionated RT to complete LN dissection following a positive SLN decreased nodal recurrence but did not improve survival (120, 121). Recent, dramatic shifts in the management of human melanoma have incorporated immunotherapy as a first line therapy for advanced melanoma (122). This reliance on immunotherapy as a cornerstone of treatment has forced a spotlight on the importance of preserving draining LNs and immune cells to promote a robust immune response (108, 109, 123, 124). Elective LN dissection or irradiation prior to checkpoint inhibitor therapy may indeed reduce the efficacy of immune checkpoint inhibitors, supporting efforts to conserve functional anti-cancer immune cells (110, 123). As anti-canine checkpoint inhibitors become incorporated into therapeutic paradigms (125–128), it will be important to critically evaluate the prognostic role of LN metastasis in patient outcomes and rely on evidence based approaches to drive the need for LN extirpation or adjuvant nodal RT in the context of effective immune responses.

Because of the evolution of staging schemes, and lack of systematic approach to staging in veterinary oncology, the proposed risk stratification scheme incorporates specific designations within the N staging category to allow for prognostication of differences in type and number of nodal spread as well as allow for presumptive conclusions to be made and transparently shared. Of note, this stratification scheme has not addressed tonsillar invasion or metastasis. Although tonsillar involvement appears rare, one study that evaluated 53 dogs with tonsillar melanoma found that 47% represented metastatic lesions from a known primary lesion, yet only 17% had concurrent lymph node metastasis (73). Therefore, a thorough oral examination should include an assessment of the tonsillar crypts when dogs are evaluated for metastasis to further examine the incidence of tonsillar spread. Cytology/biopsy of the tonsil without gross enlargement does not appear necessary at this time for complete screening, especially as the tonsil was never found to be a primary source of draining during indirect CT lymphangiography (oral SLN mapping) (73).

Distant metastatic disease was clearly found to affect prognosis, despite low study numbers and low levels of evidence to support this conclusion. Thoracic radiographs alone were used in 60% of the studies evaluated here, highlighting bias from potential under-reporting of metastasis (91, 129). While it is recommended that thoracic CT scans be utilized for screening to help better stratify risk and understand the prognostic implications of small early nodules on OMM, the use of CT adds substantial cost for some pet owners. Further, the prognostic implications of small pulmonary nodules detected by CT, but absent on radiographs, is largely unknown. Acknowledging the potential lead-time bias posed by monitoring dogs with distant metastasis detected with advanced imaging as opposed to radiographs, the proposed stratification scheme adds a designation for detection method. Despite that distant metastasis is rarely confirmed, a designation to highlight confirmation of metastasis has been included. There are also several presumptions and assumptions within the distant metastatic category that could be clarified in future versions. Two primary presumptions include investigators interpret data similarly, such as the implications of a solitary pulmonary nodule or mass, and that thoracic imaging is the likely modality used for detection of distant metastasis. The other primary assumption is that distant metastatic disease in distinct anatomic organs confers the same prognostic effect.

Strong conclusions regarding signalment, tumor localization within the oral cavity, mitotic activity, and nuclear atypia were not consistently associated with impacts on outcome. A lack of association regarding oral localization may be due to the inconsistency in the reporting process of tumor location. For example, lip tumors may include several locations, including mucocutaneous junction, haired skin of the lip, or the buccal mucosa with lesions in haired skin having a markedly different biologic behavior (75). Further, the presence of bone invasion, a strong prognostic feature, is inherently associated with location, with only lesions located on the maxillary/mandibular gingiva and alveolar mucosa able to invade the underlying bone. Most articles were not specific enough with tumor localization to separate the prognostic implications of bone invasion from oral cavity location, which may have diluted the results. Conversely, rostral versus caudal tumors are unlikely to carry uniquely different biologic behavior, but rostral tumors are theoretically easier to completely excise compared to tumors in the caudal oral cavity (13, 24, 70). Modern surgical techniques and oral surgery training opportunities increase the potential for adequate margins regardless of intraoral location, and likely contribute to the lack of prognostic influence of the tumor location.

Histopathological criteria including nuclear atypia score, mitotic count, Ki67 proliferation value, and quantification of pigmentation can also act to improve predictive algorithms needed for therapeutic decision-making (92). Because of the low level of evidence in our analysis, consistent with that of the recent consensus (75) additional data is needed to firmly assess the prognostic impact of these variables both independently and when used in combination. The risk stratification scheme includes designations to highlight tumor-specific mitotic activity and nuclear atypia. Mitotic activity includes mitotic count and Ki67 values, which could be included independently or together. Because Ki67 immunohistochemistry is not standard on many histopathologic reports and may increase cost to clients without necessarily changing a therapeutic recommendation, it may not be consistently ordered. Additionally, as with pigmentation, there is subjectivity to its interpretation, and standardized protocols and assessments across laboratories pose additional challenges (75, 92, 130–132). However, critical evaluation of the literature suggests that Ki67 may be important for prediction of biologic behavior (62). Standardized reporting of Ki67 is strongly recommended to make future conclusions on utility and clinical use. This will be especially impactful in the rare clinical case of discordant MC and KI-67 proliferative value. In this case it is suggested that both values are reported for future analysis, but Ki-67 value is adopted for scoring.

Due to the limited literature on the degree of pigmentation on prognosis for canine OMM and the inherent subjectivity of its determination, we elected to exclude pigmentation from the proposed scheme. However, we acknowledge that the degree of pigmentation is currently essential in separating HDWMN (3, 10) from OMM, based on high pigment, low mitotic index, and lack of nuclear atypia. Yet, the methods to designate oral melanocytic tumors in dogs as HWDMN or OMM have been developed but have yet to be adequately validated and concern exists surrounding if these are truly two separate entities versus a spectrum of disease. Frankly, it is unknown if HWDMN has the potential to progress to OMM, which is a critical distinction. Ultimately, these questions need to be addressed by future prospective studies. Thus, rather than continuing to refer to these two tumors as separate entities based on pathologic features and excluding HDWMN from prospective analysis, we recommend that all oral melanocytic tumors be scored using the proposed risk stratification scheme. If clinically relevant, the degree of pigmentation and differentiation of the cells can be mentioned in “additional designations.” Inclusion of all melanocytic lesions in the stratification scheme will aid in defining less biologically aggressive subset(s) of OMM, answer relevant questions on the biologic behavior of the currently termed, HDWMN, and directly influence treatment recommendations, such as the need for wide surgical margins and staging frequency in these less aggressive lesions.

In future variations of risk stratification scheme, several alterations can be made to reflect different therapeutic avenues. As immunotherapies emerge that have the potential to improve outcomes for dogs with OMM, the addition of categories such as intratumoral PD-L1, PD-1 and T lymphocyte infiltration will undoubtedly be needed (41–44, 133). Further, evaluation of the depth of invasion, also known as Breslow thickness, is a critical pathologic step in staging human melanoma (100) and may be of prognostic importance for canine OMM in the future. None of the reviewed articles described the pathologic depth of invasion limiting any conclusions on its current or future utility in dogs.

As previously stated, one limitation of the assessments made here includes the strict, pre-defined inclusion criteria, which were meant to minimize bias. Despite this, variables considered key to decision-making and development of future conclusions were similar to a recent consensus (75). A second potential limitation is that only peer-reviewed studies published in the English language were included. The authors are aware that the use of an expanded staging scheme to aid in risk stratification will increase effort. However, the ability to capture key data simply from an expanded TNM description will strengthen future efforts to form actionable conclusions regarding prognostic variables and their influence (or lack thereof) on therapeutic decision-making and outcomes.

## Data Availability

The original contributions presented in the study are included in the article/Supplementary material, further inquiries can be directed to the corresponding author.

## Footnotes

^1^ https://sciwheel.com/?lg

^2^ https://www.covidence.org
