# Supplementary material for: Risk stratification scheme based on the TNM staging system for dogs with oral malignant melanoma centered on clinicopathologic presentation
Source: Front Vet Sci. 2024 Sep 25;11:1472748. doi: 10.3389/fvets.2024.1472748 (PMC11463030; doi:10.3389/fvets.2024.1472748)
Supplement: Supplementary file 1 [file Table_1.docx]

**Supplemental Table 1: Clinical scenarios illustrating the use of the proposed risk stratification scheme and its interpretation.**

| **TNM Designation** | **Interpretation** |
| --- | --- |
| T2_a0b1c0_N3_a_M0 | Dog with a T2 oral melanoma with no evidence of osseous lysis, high mitotic activity, and low nuclear atypia that has a single metastatic lymph node with extranodal extension that was identified by SLN biopsy but no evidence of distant metastasis on CT |
| T2_a1b1c1_N3_a_M0_r_ | Dog with a T2 oral melanoma with osseous lysis, high mitotic activity, and high nuclear atypia that has a single metastatic lymph node with extranodal extension that was identified by SLN biopsy but no evidence of distant metastasis on radiographs |
| T2_a1b1c1_N3_ac_M0 | Dog with a T2 oral melanoma with osseous lysis, high mitotic activity, and high nuclear atypia that has a single metastatic lymph node with extranodal extension that was cytologically confirmed following SLN biopsy but no evidence of distant metastasis on CT |
| T3_a1b1c*_N4_i_M0 | Dog with a T3 oral melanoma with osseous lysis and high mitotic activity (nuclear atypia not reported) that has multiple presumptive positive lymph nodes identified via imaging only (not biopsy or cytology-confirmed), but no evidence of distant metastasis on CT |
| T2_a*b1c*_N1M0 | Dog with a T2 oral melanoma with high mitotic activity (bony lysis not assessed, nuclear atypia not reported) that has one regional lymph node with micro-metastasis identified following histopathologic evaluation, but no evidence of distant metastasis on CT |
| T1_a*b*c*_N0_i_M0_r_ | Dog with a T1 oral melanoma without histologic parameters identified (bony lysis not assessed, perhaps tumor diagnosed cytologically), lymph nodes negative for metastasis based on imaging alone, no evidence of distant metastasis on radiographs |
| T1_a0b0c0_N0_i_M1r | Dog with a T1 oral melanoma without evidence of osseous lysis, low mitotic activity, and low nuclear atypia that has presumptive negative lymph nodes for metastasis based on imaging alone, but evidence of distant metastatic disease that has not been cytologically or histopathologically confirmed |
| T2 _a*b*c*_N*M* | Dog with a T2 oral melanoma without histologic parameters identified (bony lysis not assessed, perhaps tumor diagnosed cytologically),that has an unknown nodal and metastatic status |
| T3_a1b1c*_N4M1_p_ | Dog with a T3 oral melanoma with osseous lysis and high mitotic activity (nuclear atypia not reported) that has multiple lymph nodes histopathologically positive for metastasis and pathologically/cytologically confirmed distant metastasis |

**Supplemental Table 2: The risk stratification scheme for dogs with oral malignant melanoma converted for use as a daily clinical worksheet**.

| **TUMOR** | **Description** | **T Stage** | **Clinical Use** |
| --- | --- | --- | --- |
| Tumor size | <2 cm | T1 |  |
|  | 2-4 cm | T2 |  |
|  | ≥ 4 cm | T3 |  |
| *Tumor designations* | | | |
| Presence of bone invasion | No | a0 |  |
|  | Yes | a1 |  |
| Mitotic activity*  Check box to indicate approach  **** Ki67 **** Mitotic Count  <19.5 <4  >19.5 ≥4  *If discordant results between Ki-67 and MC, use KI-67 for scoring | Low | b0 |  |
|  | High | b1 |  |
| Nuclear atypia | <30% | c0 |  |
|  | >30% | c1 |  |
| *Additional designations* | | |  |
| Tumor feature is unknown | | _*_ |  |
| **Tumor Stage Summary** | |  | |
| **LYMPH NODE METASTASIS** | **Description** | **N Stage** | **Clinical Use** |
| Lymph node metastasis | None | N0 |  |
|  | Micrometastasis (< 2mm) in a single node | N1 |  |
|  | Macrometastasis (> 2 mm) in a single node | N2 |  |
|  | Macrometastasis with extranodal extension in a single node | N3 |  |
|  | Multiple metastatic (micro or macro) lymph nodes | N4 |  |
| *Lymph node designations* | | | |
| LN detected by sentinel lymph node guided biopsy/cytology | | a |  |
| Metastatic disease is cytologically confirmed | | c |  |
| Metastatic disease is presumptive based on imaging only | | i |  |
| Lymph node status is unknown | | _*_ |  |
| **Lymph Node Stage Summary** | |  | |
| **DISTANT METASTASIS** | **Description** | **M Stage** | **Clinical Use** |
| Evidence of distant metastasis | Absent | M0 |  |
|  | Present | M1 |  |
| *Metastasis designations* | | | |
| Distant metastatic status is unknown | | * |  |
| Metastatic disease detected on radiographs | | r |  |
| Metastatic disease is cytologically or histologically confirmed | | p |  |
| **Distant Metastasis Stage Summary** | |  | |
| **Total Score:** | | | |
